# Danazol: An Effective Option in Acquired Amegakaryocytic Thrombocytopaenic Purpura

**DOI:** 10.1155/2015/171253

**Published:** 2015-04-05

**Authors:** E. Mulroy, S. Gleeson, S. Chiruka

**Affiliations:** Department of Haematology, Dunedin Hospital, Great King Street, Dunedin 9016, New Zealand

## Abstract

Acquired amegakaryocytic thrombocytopaenic purpura (AATP) is a rare haematological condition characterised by isolated thrombocytopaenia with normal other cell lines. It is often initially misdiagnosed as immune thrombocytopaenic purpura but has characteristic bone marrow findings of reduced megakaryocyte numbers. The optimal treatment of AATP is not clearly defined but revolves around immunosuppressive therapies. We report a case of successful treatment of AATP with danazol, an antioestrogenic medication. We also review the aetiologies and pathogenesis of the disorder and suggest that danazol should be considered as an effective alternative to potent immunosuppression in AATP.

## 1. Introduction

Acquired amegakaryocytic thrombocytopaenic purpura (AATP) is a rare haematological disorder characterised by isolated thrombocytopaenia with bone marrow amegakaryocytosis but normal other cell lines [1, 2]. Patients will often initially be misdiagnosed as having immune thrombocytopaenic purpura (ITP) but, in contrast to ITP, will usually not be steroid responsive [1–7]. Various pathogenetic mechanisms have been proposed to account for the occurrence of AATP (see Section 3), most centering around abnormalities of immune control. Treatment remains controversial and usually takes the form of immunosuppressive medications [1–9]. We report a case of AATP with sustained response to danazol therapy. Danazol has been shown in previous reports to be effective in some cases of AATP and we suggest that it remains a safe and effective medication which should be considered prior to embarking down the route of potent immunosuppression [10].

## 2. Case Report

A 65-year-old man with a history of essential hypertension, paroxysmal atrial fibrillation, dyslipidaemia, and Dukes B colon cancer 17 years previous treated with colectomy and adjuvant chemotherapy (currently in remission) was found to have a platelet count of 8 × 10^9^/L (150–400 × 10^9^/L) on a blood test in April 2012. Medications at the time included aspirin of 100 mg daily, metoprolol succinate of 95 mg daily, atorvastatin of 40 mg daily, and quinapril of 2.5 mg daily. His alcohol consumption was 8 units/week. He had noted some easy bruising and had a petechial rash. Haemoglobin and neutrophil counts were normal. Hepatitis A, Hepatitis B, Hepatitis C, HIV, EBV, and CMV serology were negative. Anti-nuclear antibodies were negative. Rheumatoid factor was in the normal range. IgM levels were mildly reduced at 0.3 g/L (0.4–2.5 g/L), but IgG and IgA levels were normal. Serum protein electrophoresis was normal. Serum-free light chains were within the normal ranges. Iron studies, vitamin B12, and folate levels were all normal. Marked thrombocytopenia, mild macrocytosis, and occasional reactive lymphocytes were seen on blood film. Given the poor response to standard therapy, CT of chest, abdomen, and pelvis was performed, looking for malignancy, but it showed no evidence of lymphadenopathy or other neoplasia.

He was initially treated for presumed immune thrombocytopaenic purpura (ITP) with oral prednisone at a dose of 1 mg/kg daily. Due to poor clinical response, intravenous methylprednisolone of 1 g/day for 3 days and intravenous immunoglobulin (0.4 g/kg/day) for 5 days were given. This led to a transient but nonsustained improvement in platelet counts (to a maximum of 132 × 10^9^/L). Bone marrow aspirate and trephine were performed, showing overall bone marrow cellularity of approximately 50%, with reduced megakaryocyte numbers (see Figures 2 and 3). There was no increase in CD34, CD117 positive cells. CD3 and CD20 stains showed no evidence of abnormal lymphoid infiltration. A diagnosis of acquired amegakaryocytic thrombocytopaenic purpura (AATP) was made.

**Figure 1:**
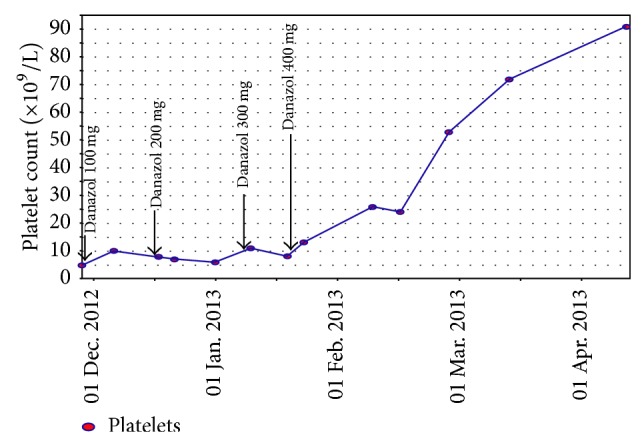
Platelet count (×10^9^/L) (*y*-axis) versus time (*x*-axis) showing dramatic improvement in platelet counts with danazol of 400 mg daily. With subsequent weaning of danazol therapy to 100 mg alternate days, platelet counts remain normal.

**Figure 2:**
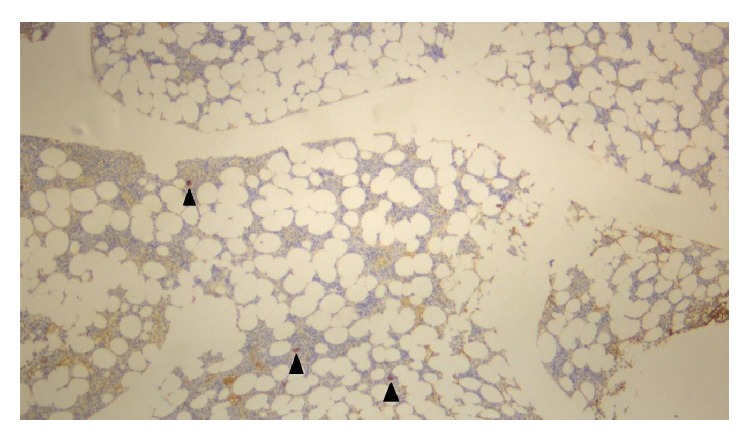
Von Willebrand stain of bone marrow trephine showing three megakaryocytes (red black arrowheads) (magnification: ×5).

**Figure 3:**
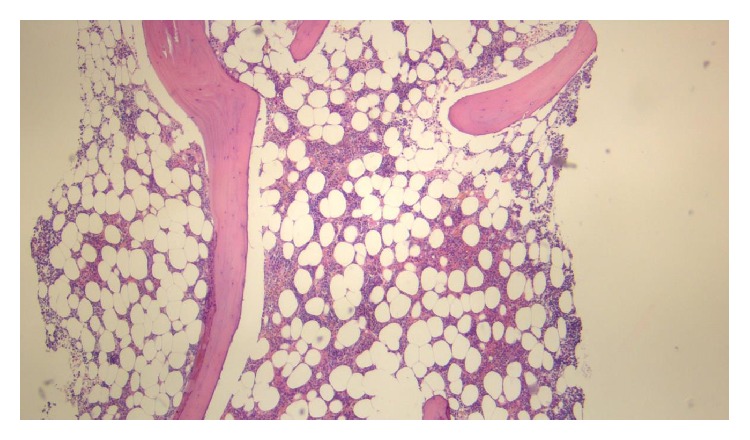
Haematoxylin and eosin stain of bone marrow trephine showing absence of megakaryocytes (magnification: ×5).

Due to the lack of sustained response to treatment with steroids and IVIG, a decision was made to commence treatment with danazol. Treatment dose began at 100 mg daily but was escalated to 400 mg daily due to lack of response to lower doses (see Figure 1). Over the following 18 months, he has successfully been weaned to a danazol dose of 100 mg on alternate days without any recurrence of thrombocytopaenia. Liver function tests have remained normal throughout therapy though he did develop manageable impotence.

## 3. Discussion

### 3.1. Background

Acquired amegakaryocytic thrombocytic purpura (AATP) is a rare haematological disorder characterised by low platelet counts, decreased or absent megakaryocytes in the bone marrow, and otherwise normal erythropoietic and myeloid cell lines [1, 2].

Isolated thrombocytopaenia is a common problem in clinical practice. There are numerous causes, including drug-induced thrombocytopaenia and ITP. Cases of amegakaryocytic thrombocytopaenia will often initially be misdiagnosed as one of these disorders.

The differential diagnosis of isolated thrombocytopaenia is wide and can broadly be divided into decreased marrow production of platelets, increased peripheral platelet destruction, idiopathic and platelet sequestration.

AATP usually presents with symptoms of excess bleeding and easy bruising [11]. A petechial rash may be present [11]. Splenomegaly is typically absent [11]. Laboratory investigations will reveal an isolated low platelet count [11]. Platelet survival studies are normal [11]. In contrast to ITP, where the number of megakaryocytes in the bone marrow is increased, cases of AATP have markedly reduced or absent bone marrow megakaryocytes [3].

### 3.2. Pathogenesis

The pathogenesis of AATP is, as of yet, unclear and there are many competing theories. AATP can be looked upon as a heterogeneous group of disorders resulting in a common downstream clinical manifestation. The finding of hypomegakaryocytosis (decreased number of megakaryocytes) or amegakaryocytosis (no megakaryocytes) in the bone marrow of patients with acquired thrombocytopaenia suggests one of the following processes.

*(1) Suppression of Megakaryocyte Maturation by an Exogenous Agent.* Viral infections, toxin exposure, nutritional deficiencies, drug ingestion, alcohol excess, and previous radiotherapy to the bone marrow can all cause hypomegakaryocytosis of varying degrees. These factors must be sought when investigating amegakaryocytic thrombocytopaenia [3, 12–14].

*(2) Suppression of Megakaryocyte Maturation by Endogenous Stimuli*.* Immune suppression* of megakaryocytes can be a primary (idiopathic) abnormality or can be secondary to other disease states affecting immune function, for example, systemic lupus erythematosus [15, 16]. Both the humoral and cellular components of the immune system may be involved. Studies have shown both humoral (IgG directed against megakaryocytes, thrombopoietin, or the thrombopoietin receptor) and cellular (T cell mediated suppression of megakaryocyte colony forming units) mechanisms contributing to the pathogenesis of AATP [17–21]. Depending on the predominant mechanism of immune-mediated amegakaryocytosis, patients may respond better to therapy targeting the humoral (e.g., rituximab [4]) or the cellular (e.g., cyclosporine [22], antithymocyte globulin [1]) immune function. It is possible that with advancement in medical knowledge we will be able to characterise patients with AATP according to their mechanism of immune amegakaryocytosis and tailor treatment appropriately, but, to date, this is not done.

*Hormonal suppression* of megakaryocyte function is associated with hyperoestrogenic states. Oestrogen has been shown to impair haematopoiesis in animals [23], to cause thrombocytopenia in humans [24], and to regulate Fc receptor expression on macrophages [25]. Administration of diethylstilbestrol has been reported to lead to amegakaryocytic thrombocytopaenia [24]. The exact mechanism of oestrogen-induced megakaryocyte suppression remains uncertain but is thought to be due to modulation of Fc receptor expression on macrophages and facilitating reticuloendothelial phagocytosis [25].

*(3) An Early Manifestation of a Stem-Cell Abnormality*. A proportion of cases of amegakaryocytic thrombocytopenia go on to develop leukaemia, myelodysplasia, or aplastic anaemia [3, 26]. This suggests an intrinsic defect in early stem cell development with hypomegakaryocytosis as an initial manifestation of the disorder.

*(4) Marrow Infiltration*. Replacements of normal marrow constituents with infiltrative or malignant processes could lead to hypomegakaryocytosis, but its occurrence in isolation without affectation of other cell lines would be extremely unusual.

### 3.3. Treatment

Reflecting the variety of pathophysiologic processes leading to AATP, many different treatments have been trialed for this disorder, with varying degrees of success. The first step in treatment is always to remove any reversible factors, for example, alcohol excess, which contribute to AATP. Monotherapy with corticosteroids, in contrast to ITP, is generally ineffective for AATP (though some groups have reported success with this approach) [8, 9]. Other treatments, mostly revolving around immunosuppression (cyclosporine A, cyclophosphamide, vincristine, IVIG, ATG, and rituximab) and splenectomy, have had varying success [1–7]. In recent times, reports of successful AATP treatment with thrombopoietin mimetics have emerged [27]. Bone marrow transplant has also been successful, especially in refractory cases [6].

A synthetic derivative of ethisterone, danazol, has been successfully used in the treatment of cyclic AATP [28]. The first report of successful use of danazol for the treatment of AATP came in 1985 [10]. It works by negative feedback on the hypothalamus leading to a hypooestrogenic and hypoprolactinaemic state. As previously mentioned, hyperoestrogenic therapies have previously been associated with impaired megakaryocyte maturation [23, 24]. Exactly how danazol improves amegakaryocytic thrombocytopenia is unknown but may relate either to Fc receptor modulation on macrophages or to its effects on oestrogen [23–25]. Side effects of danazol treatment are mainly androgenic, for example, acne, hirsutism, weight gain, and menstrual irregularities. It may also cause hepatic dysfunction [29]. In contrast to other available treatments for AATP, however, danazol has no immunosuppressive effects and should therefore be considered as a safe and cheap alternative to other therapies for this condition.

## 4. Conclusion

Acquired amegakaryocytic thrombocytopaenic purpura is a rare haematological disorder which needs to be kept in mind as a differential diagnosis in cases of immune thrombocytopaenic purpura (ITP), especially if these are not responding to conventional therapies. Bone marrow findings are characteristic, showing reduced or absent megakaryocytes with sparing of other cell lines. No single treatment has been shown to be effective in all cases of AATP. Most will not respond to steroids and IVIG alone and will often require more potent immunosuppression. We report a case of AATP responding to danazol and suggest that this treatment should be considered as a relatively safe and cheap alternative to potent immunosuppression.
